# Botanical Description of British Plants

**Published:** 1806-07

**Authors:** 


### *Botanical Description of British Plants.*

( Continued from Vol. xv. pp. 261—270. )

4. Fagus. *F. castanea.*

*Ang.* Chesnut. Chesnut-beech.

*Gen. Desc.* M. and F. fl. on the same plant. Bloss. 0. M. cal. five-cleft, bell one-shaped. Fem. cal. four-toothed, changing to a prickly four valved two-seeded capsule.

*Spec. Desc.* *Leaves* spear-shaped, long-pointed, with tapering serratures, ending in a thorn, naked underneath. *Catkins* cylindrical. *Woods, hedges.* Bl. May.

*Use.* The nuts constitute a great part of the food of the common people in the South of Europe; and hogs feeding on them, as they run wild in the forests, are reckoned particularly excellent. They are used by us in winter as an agreeable addition to our deserts. They are used for whitening

---

ening linen cloth, and for making starch.—The wood is applicable to the same uses that oak is put to: it is very durable, and some of the oldest buildings in London are said to be constructed of this wood: if the bark be not taken off, it lasts, as poles for espaliers, dead fences or hop yards, and pipes to convey water under-ground, longer than oak or elm.—Nothing will thrive under its shade.—*Withering.* At Tortworth in Gloucestershire is a chesnut, supposed to be at least 1100 years old.—*Bath. Soc. pap.* 1.

5. FAGUS. *F. sylvatica.*

*Ang.* Beech. Common beech-tree.

*Gen. Desc. As above.*

*Spec. Desc.* *Leaves* egg-shaped, indistinctly serrated. *Bark* smooth, white. *Catkins* globular. *Woods, hedges in calcareous soil.* BL. March, April.

*Use.* The nuts, or masts as they are called, when eaten, occasion giddiness and head-ache: but, well dried and powdered, they make wholesome bread. They are sometimes roasted and substituted for coffee. The poor people, in Silesia, use the expressed oil from them for butter.—The leaves gathered in autumn, before they are much injured by the frosts, make infinitely better mattrasses than straw, or chaff, and endure for 7 or 8 years.—The wood is brittle, and soon decays in the air, but endures under water. It is formed into tool-handles, planes, mallets, chairs, bedsteads, &c.; split into thin layers, it is used to make scabboards for swords: it is excellent fuel, and when burnt affords a large quantity of potash.—No verdure will flourish under the shade of this tree: it loves a rich soil; is tender when young; and is difficult to transplant: but bears lopping well, and may be trained to form very lofty hedges.—Sheep and goats eat the leaves; a horse eat them: the masts fatten swine, and are greedily devoured by mice, squirrels, and birds.—*Withering.*

### Dodecandria. Trigynia.

6. RESEDA. *R. luteola.*

*Ang. Wild woad. Dyer's weed. Dyer's yellow weed. (Wolds, Woulds, Welds.)*

*Gen. Desc.* Cal. one-leaf, divided. Petals jagged. Caps. one-celled, many-seeded, opening at top.

*Spec. Desc.* *Leaves* strap-spear-shaped, with a minute reddish tooth on each side the base. *Cal.* four-cleft, spear-shaped, two upper wide asunder. *Stem.* cylindrical hollow, furrowed. *Petals* three, upper-hand-shaped, four-div. two lateral oblong, cloven. *Nectary* broad, hollow without at the the base, and covered with a thin concave lid. *Stam.* 20 to 30, or more. *Germ.* pyramidal, three-sided, blunt. *Styles.* 0. *Summits.* 3. *Caps.* three-valv. rolled so as to enfold the seeds. *Flowers* yellow. *Meadows, pastures, walls.* Bl. June, July.

*Use.* This plant affords a most beautiful yellow dye for cotton, woollen, mohair, silk and linen; and is that which is most commonly used by the dyers for that purpose, as it gives the brightest dye. Blue cloths, dipped in a decoction of it, become green. The yellow colour of the paint called Dutch pink, is got from this plant. The tinging quality resides in the stems and roots; and it is cultivated in sandy soils, because rich soil is apt to lessen its value by making the stalk hollow.—Cattle will not eat it, but sheep sometimes browse it a little.—*Withering.*

7. Euphorbia. *E. Characias.*

*Ang.* Red spurge.

*Gen. Desc.* Cal. one-leaf, distended. Bloss. four or five petals, sitting calyx. Caps. three, united.

*Spec. Desc.* *Umbels* with many short forked spokes; small crowded. *Invollucell.* perforated, cloven. *Stem.* shrub-like, four feet high, woolly. *Leaves* entire, spear-shaped, leather-like, downy, reflected, green, an elevated rib on each side. *Flowers* within the first invollucell. male, the rest hermaph. v. dark purple. *Germens,* very woolly. *Floral-leaves* bend back. *Woods, hedges, rare.* Bl. June.

*Use.* The powdered leaves, in doses of 15 to 25 grains, operate as a purge. The juice of every species of Spurge is so acrid, that it corrodes and ulcerates the body wherever it is applied; so that Physicians have seldom ventured to use it internally. Warts or corns anointed with the juice, presently disappear. A drop of it put into the hollow of a decayed and *aching tooth* destroyes the nerve, and consequently removes the pain. Some people rub it behind the ears, that it may blister, and by that means give relief. *Withering.*

Dodecandria. Dodecagynia.

8. Sempervivum. *S. tectorum.—Sedum majus vulgare.*

*Ang.* Houseleek. Cyphel.

*Gen. Desc.* Cal. twelve-cleft. Petals. 1, 6, 12, to 24. Caps. 12, many seeded, like a legumen.

*Spec. Desc.* *Leaves* fleshy, fringed; offsets expanding. *Flowering-branches* bowed back. *Petals* smooth within; fringed with pellucid hairs, pale red. *Stam.* 6 to 25. *Pis-*

*tils*

*tils* 12, in an oval or triangular form. *Roofs, old walls.* Bl. July.

*Use.* The juice, either applied by itself or mixed with cream, gives present relief in *burns* and other external inflammations. Mixed with honey, it is an useful application in *apthous* cases.—*Withering.*—It is recommended as a cooler, by way of cataplasm, to *burns* and *hot-ulcers*: and the juice, mixed with honey and laid on with a pencil, has been of service to cure the *thrush* in children. Boerhaave found that 10 oz. of the juice given internally, was beneficial in *dysenteries*, and it has been found useful in *gonorrhœas* by others.—*Lightfoot.*

### Class XII. Icosandria.

### Icosandria. Monogynia.

1. Prunus. *P. padus.*—*Cerasus racemosa sylvestris.*

*Ang.* Birds cherry. Bird cherry. Wild cluster-cherry.

*Gen. Desc.* Cal. five-cleft, beneath. Petals 5. Drupa one-celled, closed at top. Nut with seams.

*Spec. Desc.* *Flowers* in bunches, below the leaves. *Leaves* deciduous, two glands at the base underneath. *Petals* and *Cal.* finely serrated. *Stam.* 25. *Woods, hedges.* Bl. May.

*Use.* The bark is made use of by the inhabitants of Finland in a strong decoction for the cure of *venereal* complaints; which practice is corroborated by the testimony of M. Broerland: he directs 6 oz. of the dry, or 8 of the fresh bark, to be boiled in eight to four pints of water, the dose is 4 oz. four times a day; it alone cures the slighter infections, and combined with mercury facilitates the cure of the severer states of the disease.—*Venel.* A decoction of the berries is sometimes given with success in the *dysentery.*—The fruit is nauseous; but bruised and infused in wine or brandy, it gives an agreeable flavor.—The wood being smooth and tough is made into handles for knives and whips.—It grows well in woods, groves, or fields, but not in a moist soil: it bears lopping, and suffers the grass to grow under it. Sheep, goats and swine eat it. Cows are not fond of it; horses refuse it.—*Withering.*

( To be continued. )

